# Identification of dentinogenic cell-specific surface antigens in odontoblast-like cells derived from adult dental pulp

**DOI:** 10.1186/s13287-019-1232-y

**Published:** 2019-04-27

**Authors:** Kyung-Jung Kang, Chun-Jeih Ryu, Young-Joo Jang

**Affiliations:** 1 Department of Nanobiomedical Science and BK21 PLUS Global Research Center for Regenerative Medicine, Dankook University, Cheonan, 31116 South Korea; 2 Department of Integrative Bioscience and Biotechnology, Institute of Anticancer Medicine Development, Sejong University, Seoul, 05006 South Korea

**Keywords:** Adult stem cells, Dental pulp cells, Odontoblasts, Dentinogenic differentiation, Decoy immunization, Cell surface antigens

## Abstract

**Background:**

Odontoblast is a unique progenitor that plays a role in dentin formation. So far, the dentinogenic differentiation of dental pulp stem cells and the role of surface molecules of odontoblasts in dentinogenesis are not well known yet. In this study, we obtained odontoblast-like cells from human dental pulp cells and screened odontoblast-specific cell surface antigens by decoy immunization.

**Methods:**

Through decoy immunization with intact odontoblast-like cells derived from human dental pulp cells, we constructed 12 monoclonal antibodies (mAbs) of IgG type, and their binding affinities for cell surface of odontoblast-like cells were analyzed by flow cytometry. Immunoprecipitation, mass spectrometry, and immunohistochemistry were performed to demonstrate odontoblast-specific antigens. Odontoblasts were sorted by these mAbs using magnetic-activated cell sorting system, and their mineralization efficiency was increased after sorting.

**Results:**

We constructed 12 mAbs of IgG type, which had a strong binding affinity for cell surface antigens of odontoblast-like cells. In human adult tooth, these mAbs accumulated in the odontoblastic layer between dentin and pulp and in the perivascular region adjacent to the blood vessels in the pulp core. Cell surface expression of the antigenic molecules was increased during odontogenic cytodifferentiation and decreased gradually as dentinogenic maturation progressed. Proteomic analysis showed that two representative antigenic molecules, OD40 and OD46, had the potential to be components for cell adhesion and extracellular matrix structures.

**Conclusion:**

These results suggest that mAbs will be useful for detecting and separating odontoblasts from the primary pulp cells and other lineage cells and will provide information on the structures of extracellular matrix and microenvironment that appears during the dentinogenic differentiation.

**Electronic supplementary material:**

The online version of this article (10.1186/s13287-019-1232-y) contains supplementary material, which is available to authorized users.

## Background

The human dental pulp has a stem cell population with the ability to differentiate into various cell types, including neuronal cells, adipocytes, and odontoblasts [1, 2]. Much evidence has supported that postnatal dental pulp stem cells (DPSCs) isolated from the pulp tissue of human adult permanent tooth were useful in the repair of bone, cartilage, and the dental pulp itself [3]. Multiple signaling pathways, including Wnt, TGFβ/BMP, and FGF signaling, are involved in regulating whole tooth development [4, 5]; however, the dentinogenic mechanism that determines the odontogenic fate in dental mesenchyme is a complex process and remains largely unknown. BMP2 induces dental pulp cell differentiation into odontoblast lineages [6–8]. BMP2 conditional knockout mice display the delayed odontoblast differentiation, abnormal dentin tubules, and decrease expression of tooth-related genes [9, 10], and BMP2 knockout dental papilla mesenchymal cells in murine are defective in odontoblastic differentiation and odontogenesis [11]. Transcription of the dentin-specific genes is mediated by Dlx3/Osx signaling pathway in odontoblasts, which are downstream targets of BMP-2 signaling in osteogenic cells [12, 13]. BMP4 is also essential for the progression of tooth development. Previous studies with knockout mice revealed that the arrest of tooth development caused by the deletion of Msx1 and Pax9 proteins is rescued by BMP4 overexpression [14, 15], and BMP4 signaling converges on Wnt activation during odontogenesis [16]. Both collagen and non-collagenous proteins are produced during odontogenic differentiation. Among non-collagenous proteins, dentin sialophosphoprotein (DSPP), osteocalcin, osteopontin, and dentin matrix protein-1 (DMP-1) are expressed. Both DMP-1 and DSPP play a role in early stages of odontogenesis and the mineralization of dentin [17]. Cells can be isolated using proper cell surface antigens to overcome the heterogeneity and lack of reproducibility that frequently appear during the use of adult stem cells and primarily differentiated progenitors. Previously, DPSCs have been isolated using STRO-1, CD105, or CD90 [18–20]. Additionally, stem cell-specific surface markers from dental pulp have been searched for, and hDPSCs were purified using these markers [21, 22]. Dentin formation offers attractive perspectives for elaborate tooth regeneration and treatment, which enables the cure of tooth loss and restores the quality of life of the patients. Although isolation of odontoblasts from the pulp tissue must be achieved as a key element for efficient treatment, more specific cell surface markers for odontoblast identification are needed. In this report, we induced odontoblastic differentiation with human dental pulp cells (hDPCs) by BMP2/4 treatment and obtained odontoblast-like cells in the expanded culture. Through direct immunization with intact odontoblasts, we identified several candidate antibodies recognizing odontoblast-specific cell surface antigens.

## Methods

### Cell culture and chemical treatment

To primary culture of human dental pulp cells (hDPCs) and periodontal ligament cells (hDPLCs), third molar teeth were collected from dental surgery patients aged 19–29 years old under guidelines approved by the Dankook Dental Hospital, and the informed consent for all experiments using extracted teeth was obtained from all participants. Tissues were separated from the tooth and digested by 4 mg/ml dispase (Sigma-Aldrich) and 3 mg/ml collagenase type I (Millipore) for 1 h at 37 °C. Single cell suspension isolated from pulp tissue was incubated with α-MEM (Hyclone) containing 20% fetal bovine serum (Hyclone), mycoplasma removal agent (Capricorn Scientific), and antibiotics (Lonza) at 37 °C in 5% CO_2_. For induction of cytodifferentiation, hDPCs were treated with 100 ng/ml FGF, 50 ng/ml TGFβ-1 (Sino Biological), 10–100 ng/ml BMP2 (Sino Biological), and 10–100 ng/ml BMP4 (Prospec). Human fetal osteoblast (hFOB) was cultured with DMEM/F-12 (Thermo) including 10% FBS at 34 °C in 5% CO_2_. For induction of osteoblastic differentiation, hFOB1.19 (ATCC, CRL-11372) cultured at 34 °C was transferred at 39 °C. For osteogenic induction of hPDLCs, cells were treated with 100 ng/ml BMP2 (Sino Biological) for 7 days. For mineral formation, cells were incubated in media containing the mineralization additives, which consist of 100 μM ascorbic acid, 100 nM dexamethasone, and 5 mM β-glycerophosphate.

### Construction of mAbs against cell surface molecules of odontoblast-like cells

Production of mAbs against cell surface antigens was performed as previously reported with modification [22] and represented in Fig. 2A. Animal study protocol for antibody production was approved by the Institutional Animal Care and Use Committee of Dankook University. Briefly, cells were dissociated by using enzyme-free dissociation solution (Millipore), and 5 × 10^5^ cells in 30 μl PBS (pH 7.4) were injected into the hind footpads of 11 female BALB/c mice for immunization. To generate a panel of hybridomas producing antibodies that bound to the odontoblasts, odontoblasts and hDPCs were injected into the left and right hind footpads, respectively. After eight times of repeated alternate immunization, a lymphocyte suspension from the left popliteal lymph nodes was fused to FO myeloma cells (ATCC). Hybridomas were cultured in DMEM supplemented with 20% FBS (Hyclone) and HAT component (Sigma-Aldrich), and the clonal selection was performed by enzyme-linked immunosorbent assay (ELISA) and flow cytometric analysis on the hDPCs or odontoblasts.

### Immunophenotyping

Cells dissociated by enzyme-free dissociation solution (Millipore) were incubated with proper antibodies or hybridoma supernatants in PBS containing 1% BSA on ice, followed by treatment with FITC-conjugated anti-mouse IgG (1:100, Santa Cruz) as the secondary antibody. Cells were analyzed by flow cytometry in FACSCalibur™ (BD Biosciences). Antibody binding affinity was quantified by using WinMDI program.

### Antibody isotyping and antibody gene sequencing

The immunoglobulin isotype of each mAbs was determined by the Mouse Immunoglobulin Isotyping Kit (BD Pharmingen), according to the supplier’s protocol. Rat anti-mouse IgG1, IgG2a, IgG2b, IgG3, IgM, IgA, Igκ, and Igλ were used for coating multi-well plate, and hybridoma supernatant was applied into each well. The reference immunoglobulin mixtures (BD Biosciences) were used as positive controls. HRP-labeled rat anti-mouse immunoglobulin was added into each well, and the isotype signals were determined by optical density of 450 nm. For antibody gene sequencing, total RNA was extracted from hybridoma cells by Easy-spinTM Total RNA Extraction kit (Intron), and cDNA was synthesized by Maxime RT-PCR PreMix Kit (Intron). To sequence variable regions of antibody heavy and light chains, PCR primers were synthesized and used as described previously (Additional file 1: Table S1) [23]. PCR amplification of variable heavy and light chain genes was performed as followed: 1 cycle of 5 min at 94 °C, 30 cycles of 1 min at 94 °C, 1 min at 45 °C, 1 min at 72 °C, and the final cycle of 5 min at 72 °C. For heavy chain sequencing, two variable heavy chain forward primers were combined with an isotype-specific constant region reverse primer. For light chain sequencing, three κ variable light chain forward primers were combined with the corresponding constant region reverse primer. The PCR products were cloned into pBluescript KS(+) vector, and sequencing was proceeded.

### Quantitative real-time PCR

cDNA for quantitative real-time PCR (qRT-PCR) was synthesized by using the ReverTra Ace™ qPCR RT kit (Toyobo). The qRT-PCR was performed by using iTaq™ Universal SYBR Green Supermix (Bio-Rad) system. Used primers are listed in Additional file 1: Table S2. The cycling parameters of qPCR were followed: 1 cycle for 30 s at 95 °C, 40 cycles for 15 s at 95 °C, and 1 min at 60 °C. During PCR, a dissociation curve was constructed in the range of 65 to 95 °C. The GAPDH was used as an internal control to normalize the variability in target gene expression. Primer information was mentioned in Additional file 1: Table S2. Statistical analyses on three readings were carried out using Student’s *t* test, and *p* values of less than 0.05 were considered significant.

### Western analysis and immunoprecipitation

Cells were lysed using 1% NP-40 buffer (20 mM Tris-HCl, pH 8.0, 150 mM NaCl, 2 mM EGTA, 2 mM EDTA, 1% NP-40, phosphatase/protease inhibitors). For western blot analysis, the lysates were separated on SDS-PAGE, transferred to a PVDF membrane (Millipore), and then probed with antibodies against DSPP, DMP-1, Smad1, p-Smad1/5/9, Smad3, p-Smad2/3, p-p38 (purchased by Cell Signaling), p38, ERK, and p-ERK (purchased by Santa Cruz), followed by treatment with IgG-HRP (Millipore). For immunoprecipitation of surface antigens, intact cells were labeled by EZ-Link Sulfo-NHSLC-Biotin (Thermo Scientific). Biotin-labeled cell extract was incubated with antibody, followed by pull-down with Protein G-agarose (Incospharm). The immunoprecipitants were separated SDS-PAGE, transferred to a PVDF membrane (Millipore), and then probed with streptavidin (Sigma). The antigenic molecules were visualized by using ECL Western Blotting Detection Kit (GE healthcare) on film or directly by Coomassie Brilliant Blue staining.

### Immunohistochemistry

Human dental pulp tissue extracted from the third molar was fixed in 4% paraformaldehyde and was incubated in 30% sucrose, after washing with PBS. Tissue was embedded in Tissue-Tek O.C.T (Optimal Cutting Temperature) Compound (Sakura Finetechnical Co) and cut into 10-μm-thick coronal sections. Endogenous peroxidase activity was inhibited by incubation with 0.3% H_2_O_2_ in PBS for 30 min. The sections were incubated at RT for 1 h in blocking solution (5% goat serum in PBS containing 0.1% Tween 20; 0.1% PBST) and treated with the antibody at 4 °C for 16 h. Then, tissues washed for 0.1% PBST and incubated with biotin-conjugated anti-mouse IgG (Vector Laboratories) at RT for 1 h. After washing, tissue sections were incubated with VECTASTAIN ABC Reagent (Vector Laboratories) at RT for 30 min and were incubated with the DAB substrate for the development of signals. Nucleus was detected by hematoxylin and eosin staining. Microscope slides were mounted in Eukitt quick-harder mounting medium (Sigma-Aldrich), and tissues were detected by the Upright FL microscope, Nikon Eclipse 80i (Nikon).

### Magnetic-activated cell sorting

Cell sorting was performed using the magnetic-activated cell sorting (MACS) kit (Miltenyi Biotec) according to the manufacturer’s instructions. Cells were dissociated by enzyme-free dissociation solution dissociation buffer (Millipore) and incubated with 100 μl biotin-conjugated mAbs. Subsequently, cells were incubated with MACS MicroBeads (Miltenyi Biotec) and applied into a MACS column (Miltenyi Biotech), which placed in a MACS separator (Miltenyi Biotech). The mAb-positive cells were retained on the column, but negative cells were eluted in the flow-through fraction. For elution of positive fraction, the column was removed from the magnetic separator.

## Results

### Dentinogenic differentiation of human adult dental pulp cells by co-treatment of BMP2 and BMP4

**Fig. 1:**
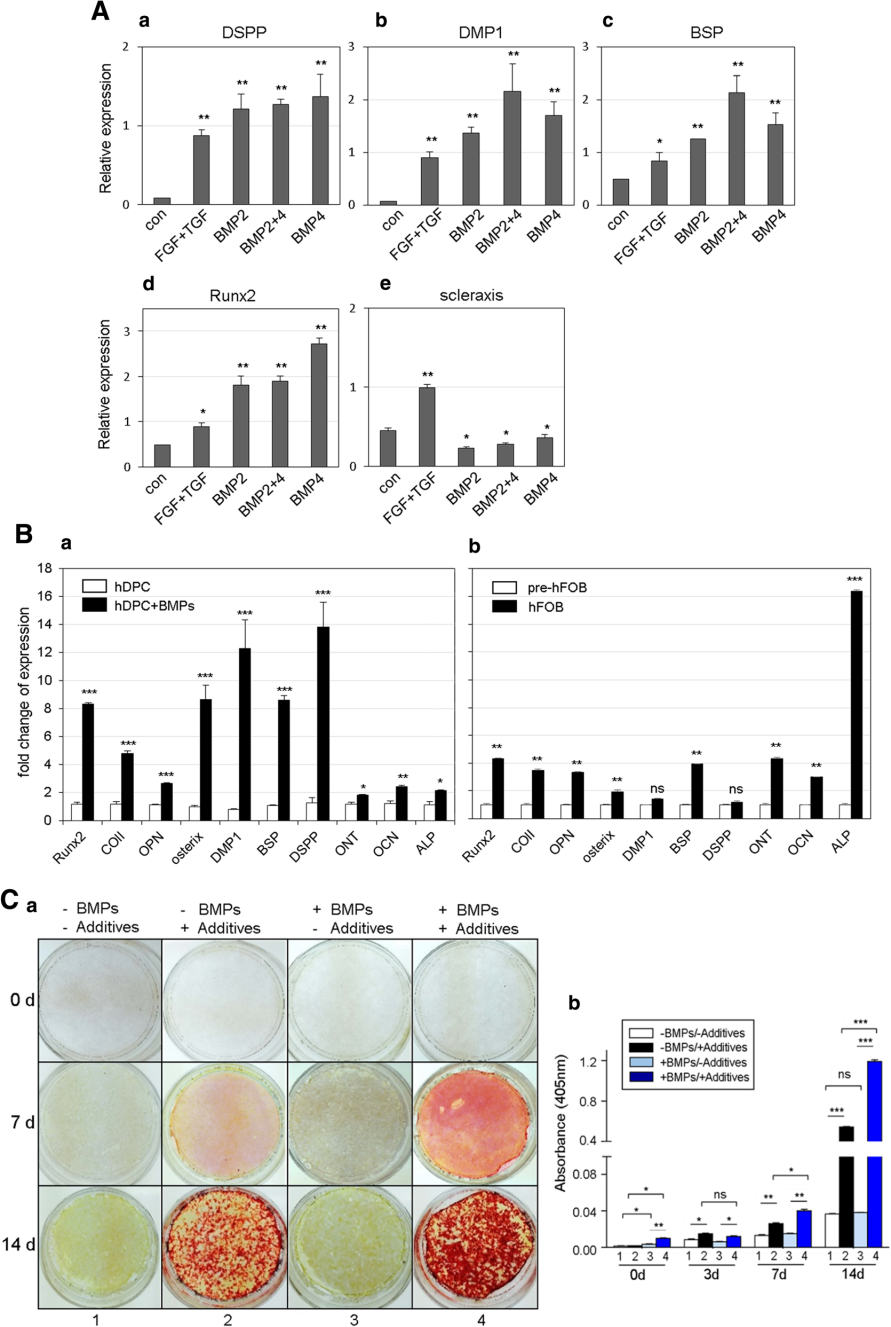
Co-treatment of BMP2 and BMP4 promotes the expression of odontoblast marker genes in hDPCs by induction of odontogenic cytodifferentiation. **A** Expression patterns of odontogenic and osteogenic markers by treatment with various cytokines. hDPCs were treated with 100 ng/ml FGF2 and 50 ng/ml TGFβ-1 (FGF+TGF), 100 ng/ml BMP2 (BMP2), 100 ng/ml BMP4 (BMP4), or 100 ng/ml BMP2 and 100 ng/ml BMP4 (BMP2+4). No treatment sample was indicated as con. **B** Comparison of expression patterns of odontogenic and osteogenic markers in between odontoblast-like cells (**a**) and osteoblasts (**b**) by co-treatment with BMP2 and BMP4. hDPC, human dental pulp cells no-treated with BMPs; hDPC+BMPs, human dental pulp cells co-treated with BMPs; pre-hFOB, human fetal osteoblasts grown at 34 °C; hFOB, human fetal osteoblasts grown at 39 °C for induction of osteo-maturation. **C** Osteogenic/dentinogenic maturation efficiency is enhanced in hDPCs treated with BMP2 and BMP4. To induce mineral formation, cells were cultured in media containing mineralization additives for the indicated time, following BMPs treatment. Mineral formation on the cell surfaces was investigated by alizarin red S staining (**a**) under the microscope and was quantified as optical density at 405 nm (**b**). −BMPs/−Additives, hDPCs untreated; −BMPs/+Additives, hDPCs incubated in media containing mineralization additives; +BMPs/−Additives, hDPCs treated with BMPs for odontogenic differentiation; +BMPs/+Additives, hDPCs incubated in media containing mineralization additives following BMPs treatment. Bar graphs represented the mean of three independent experiments ± SD. Statistical data were analyzed by Student’s *t* test, and asterisk indicated the significant difference. ***, *P* < 0.001; **, *P* < 0.01; *, *P* < 0.05; ns, not significant

To identify the optimal conditions for dentinogenic differentiation, human adult dental pulp cells were treated with various cytokines. TGFβ-1 reportedly promotes odontoblastic differentiation of dental pulp cells, and its effect was synergistically increased by FGF2 [24, 25]. When hDPCs were co-treated with FGF2 and TGFβ-1 in this study, the expression levels of odontogenic and osteogenic markers were increased compared to the controls (Fig. 1A, FGF+TGF in a–d). BMP2 treatment increased the expression levels of DSPP, DMP-1, BSP, and Runx2 by 14.1, 19.4, 2.6, and 3.8 times, respectively (Fig. 1A, BMP2 in a–d). In addition to BMP2, when hDPCs were treated with BMP4, the expression levels of DSPP, DMP-1, BSP, and Runx2 increased by 16.0, 24.3, 3.2, and 5.7 times, respectively (Fig. 1A, BMP4 in a–d). To investigate whether two BMPs exert a synergistic effect, hDPCs were co-treated with BMP2 and BMP4 (each 100 ng/ml). Consequently, the expressions of DMP-1 and BSP increased to a greater extent than the individual treatments (Fig. 1A, b and c). The expression of scleraxis, a marker of ligament differentiation, was induced by co-treatment with FGF2 and TGFβ-1 and was decreased by BMP treatment (Fig. 1A, e).

To find optimal concentrations of BMP2 and BMP4 in dentinogenic differentiation, hDPCs were treated with a combination of various concentrations of two BMPs. The co-treatment of cells with 100 ng/ml BMP2 and 10 ng/ml BMP4 increased the mRNA expressions of genes to the highest levels (Additional file 1: Figure S1). To observe the difference of gene expressions during dentinogenesis and osteogenesis, two progenitor cells were prepared as followed: odontoblast-like cells were derived from hDPCs by co-treatment with BMP2 and BMP4 in a ratio of ten to one (100 ng/ml BMP2 and 10 ng/ml BMP4) according to previous results, and osteoblasts were derived from pre-osteoblasts by incubation at restrictive temperature. In odontoblast-like cells, Runx2 and osterix levels were increased by 7.2 and 8.9 times than in dentinogenic induction, respectively (Fig. 1B, a). Similarly, DMP-1, DSPP, and BSP levels were highly increased by 16.0, 11.3, and 8.5 times in odontoblast-like cells, respectively (Fig. 1B, a). Although expression of alkaline phosphatase (ALP) was remarkably high in osteoblasts, no significant increase of expression of two dentin markers, DSPP and DMP-1, was observed in osteoblasts (Fig. 1B, b). These data indicate that the specific concentrations of BMP2 and BMP4 could maximize the efficiency of the dentinogenic differentiation of hDPCs and that the dentinogenic differentiation was totally discriminated with osteogenic differentiation in gene expressions. The phosphorylation of Smad1/5/9 was induced by BMP2 or BMP4 and increased further with co-treatment with two BMPs (Additional file 1: Figure S2), suggested that the co-treatment of hDPCs with BMP2 and BMP4 continue to activate the canonical BMP signaling at higher levels.

To investigate the mineralization efficiency, cells were incubated in media containing mineral-forming additives for the indicated times. hDPCs showed significantly less stained by alizarin red than odontoblast-like cells did after 7 days of mineralization induction (Fig. 1C, middle panels in 2 and 4 in a). After 14 days of mineralization induction, odontoblast-like cells showed a further darkened by alizarin red staining than hDPCs (Fig. 1C, lower panels in 2 and 4 in a and 14d in b). Although mineralization generally occurred after 14 days in hDPCs treated with additives, in case of odontoblast-like cells, mineral deposits were observed within 7 days of induction (Additional file 1: Figure S3). Like mineralization, ALP activity was increased by 1.3 times in odontoblast-like cells than in hDPCs (Additional file 1: Figure S4). These results suggested that co-treatment with BMP2 and BMP4 induce dentinogenic differentiation of hDPCs.

### Construction of a set of monoclonal antibodies against cell surface molecules of odontoblast-like cells

**Fig. 2:**
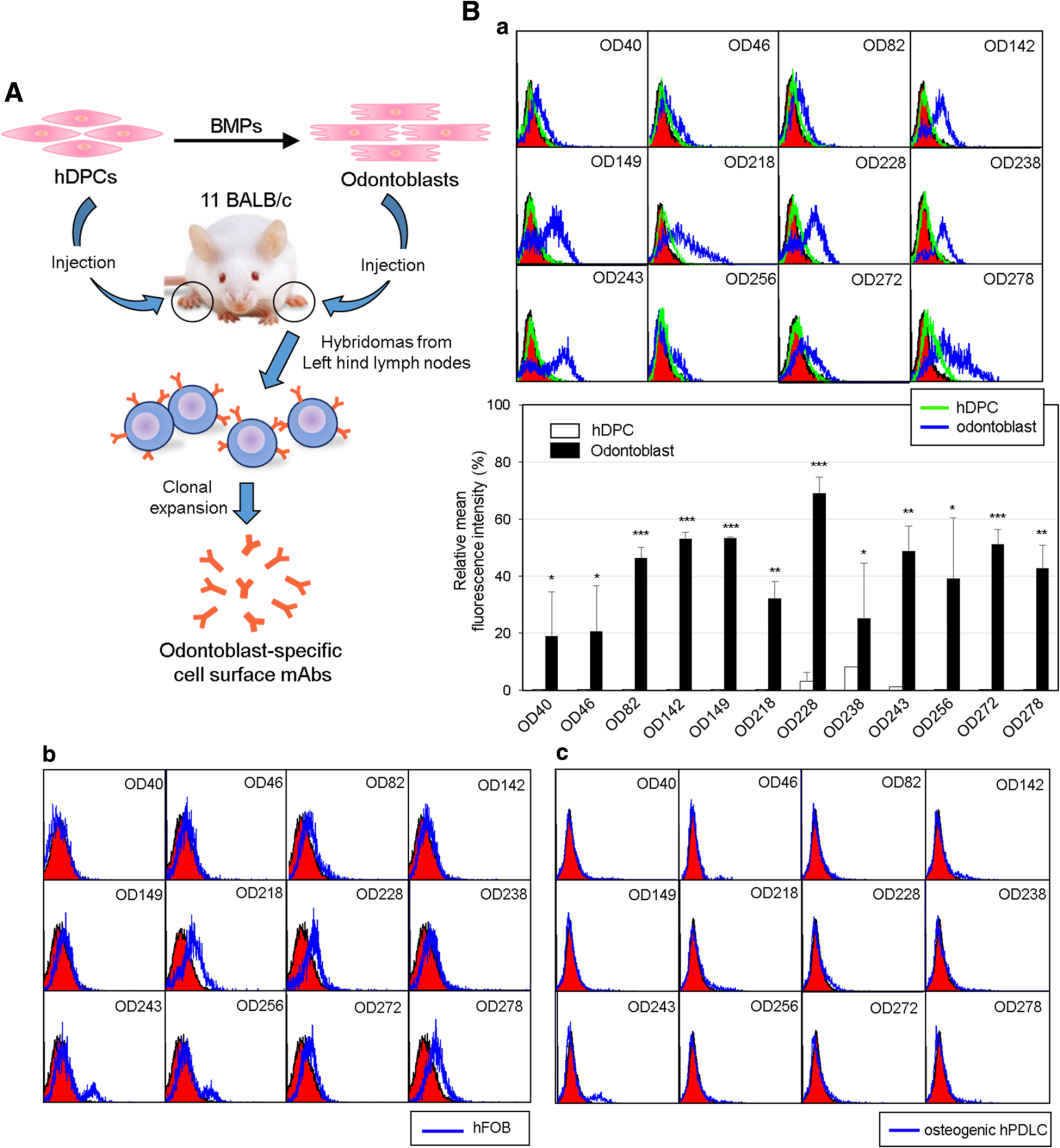
Construction of a set of monoclonal antibodies against cell surface molecules of odontoblast-like cells. **A** Schematic strategy of decoy immunization for construction of the odontoblast-specific cell surface antibodies. Strategy of antibody screening was described in the Materials and Methods. The intact 1 × 10^6^ of odontoblast-like cells and hDPCs were alternatively injected into hind foot pads of 11 BALB/c mice. After immunization, the left hind lymph nodes were subjected for hybridoma construction. **B** Cell surface binding of 12 monoclonal antibodies on odontoblasts (**a**), osteoblasts (**b**), and osteogenic hPDLCs (**c**). The interaction between each antibody and cells were analyzed by immunocytometric analysis using by FACS. The filled curves indicated a negative control, which was incubated with the secondary antibody without the primary antibody. In **a**, black and gray curves indicated the binding on odontoblasts and hDPCs, respectively. The intensities of antibody interactions were represented as mean fluorescence. Statistics of mean fluorescence values from three independent binding tests were analyzed by Student’s *t* test, and asterisk indicated the significant binding difference between hDPCs and odontoblasts. ***, *P* < 0.001; **, *P* < 0.01; *, *P* < 0.05. In **b**, pre-osteoblastic and mature osteoblastic stages of hFOB were subjected to the incubation with the primary antibodies. Filled and open curves indicated the Ab binding on pre-osteoblasts and mature osteoblasts, respectively. In **c**, filled and open curves indicated the Ab binding on undifferentiated and osteogenic hPDLCs, respectively

**Fig. 3:**
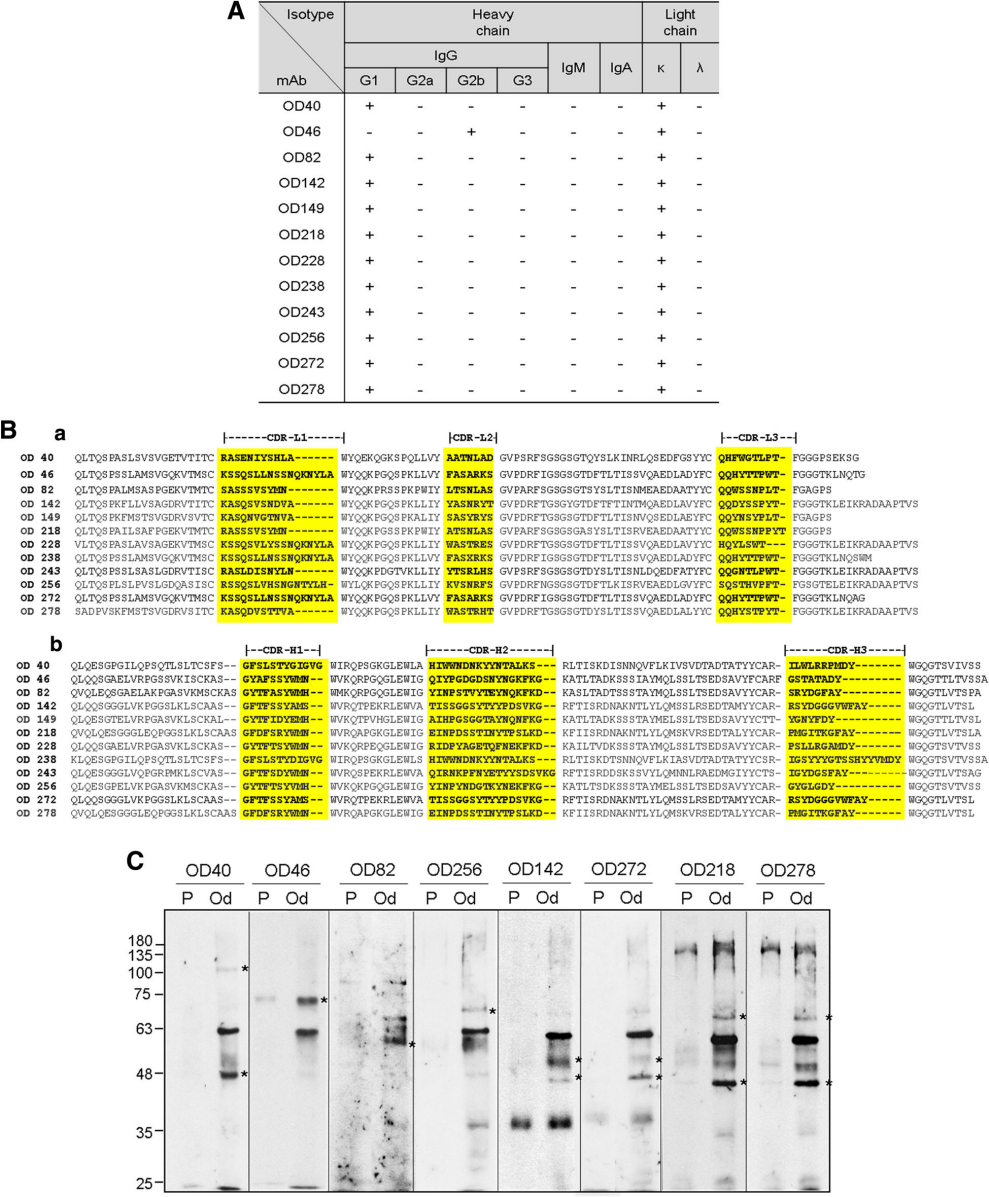
Characterization of 12 IgG-type monoclonal antibodies. **A** Isotypes of 12 mouse IgG-type monoclonal antibodies (IgG1, IgG2a, IgG2b, IgG3, and kappa/lambda) derived from hybridoma supernatant of the 12 clones. **B** Amino acid sequences of mouse immunoglobulin heavy and light chain variable regions of 12 mAbs. PCR fragments of heavy and light chain variable region genes were amplified from cDNA derived from hybridoma clones by using IgG variable region-based degenerate PCR primers as shown in Additional file 1: Table S1. The consensus sequences of IgG light chain (**a**) and IgG heavy chain (**b**) of variable regions were compared. Three complementarity determining regions in light and heavy chains (CDR-L1-3 and CDR-H1-3) were shown in shading boxes. **C** Identification of the antigenic molecules recognized by representative mAbs. Cell surface were biotinylated and antigenic molecules were captured by mAb. These molecules were visualized by western blot using streptavidin-HRP

For screening of the cell surface markers expressed specifically in odontoblast-like cells, we constructed hybridomas secreting monoclonal antibodies that specifically recognized odontoblast-like cells using an immunomic approach (see the “Methods” section, Fig. 2A). A total of 342 hybridomas were primarily selected. To confirm whether the monoclonal antibodies were specific for the odontoblast-like cells which were induced from hDPCs by treatment with BMPs, cell binding affinities were analyzed by flow cytometry. Twenty-nine of the mAbs bound to odontoblast-like cells more specifically than they did to hDPCs, and 17 and 12 of total 29 mAbs turned out to be IgM and IgG types, respectively. Only IgG types were studied further in this study. Eight mAbs appeared to be more specific to odontoblast-like cells (OD142, OD149, OD218, OD228, OD238, OD243, OD272, and OD278); of these 12, four clones bound much more weakly (OD40, OD46, OD82, and OD256) (Fig. 2B, a). To compare the antibody binding affinity in odontoblasts and osteoblasts, cell bindings of the mAbs on the hFOB and the osteogenic hPDLCs were investigated. The osteogenic differentiation of hPDLCs was confirmed by expression of the osteogenic markers (Additional file 1: Figure S5). Cell binding affinities of the mAbs for both osteoblasts were generally lower than they were for odontoblast-like cells (Fig. 2B, b and c). In addition, these antibodies were not interacted with fibrogenic and epithelial osteosarcoma cell lines such as MG63 and Saos-2, respectively (Additional file 1: Figure S6, a & b). These results suggested that 12 mAbs constructed in this study specifically recognize odontoblast-like cells. Immunoglobulin subclass of the 11 IgG clones was the IgG1 type, and the OD40 clone was the IgG2b type. All hybridoma clones contained the kappa-type light chain (Fig. 3A). In all cases, the core sequences in three CDRs of the heavy and light chains were conserved (Fig. 3B). Interestingly, OD142 and OD272 or OD218 and OD278 contained the same amino acid sequences of the heavy chain CDR regions (Fig. 3B, b), whereas their light chain CDR regions were completely different (Fig. 3B, a). To detect the antigenic molecules recognized by mAbs, we performed immunoprecipitation with biotin-labeled hDPCs and odontoblastic cells using the representative antibodies (Fig. 3C). Interestingly, the antigenic molecules immunoprecipitated by OD142 and OD272 or OD218 and OD278 were considered as the same molecules in odontoblast-like cells, respectively. Together with CDR sequences, these data suggested that these antibodies recognized the different epitopes of the same antigenic molecules.

### Monoclonal antibodies recognize the odontogenic differentiation of hDPCs in vivo and in vitro

**Fig. 4:**
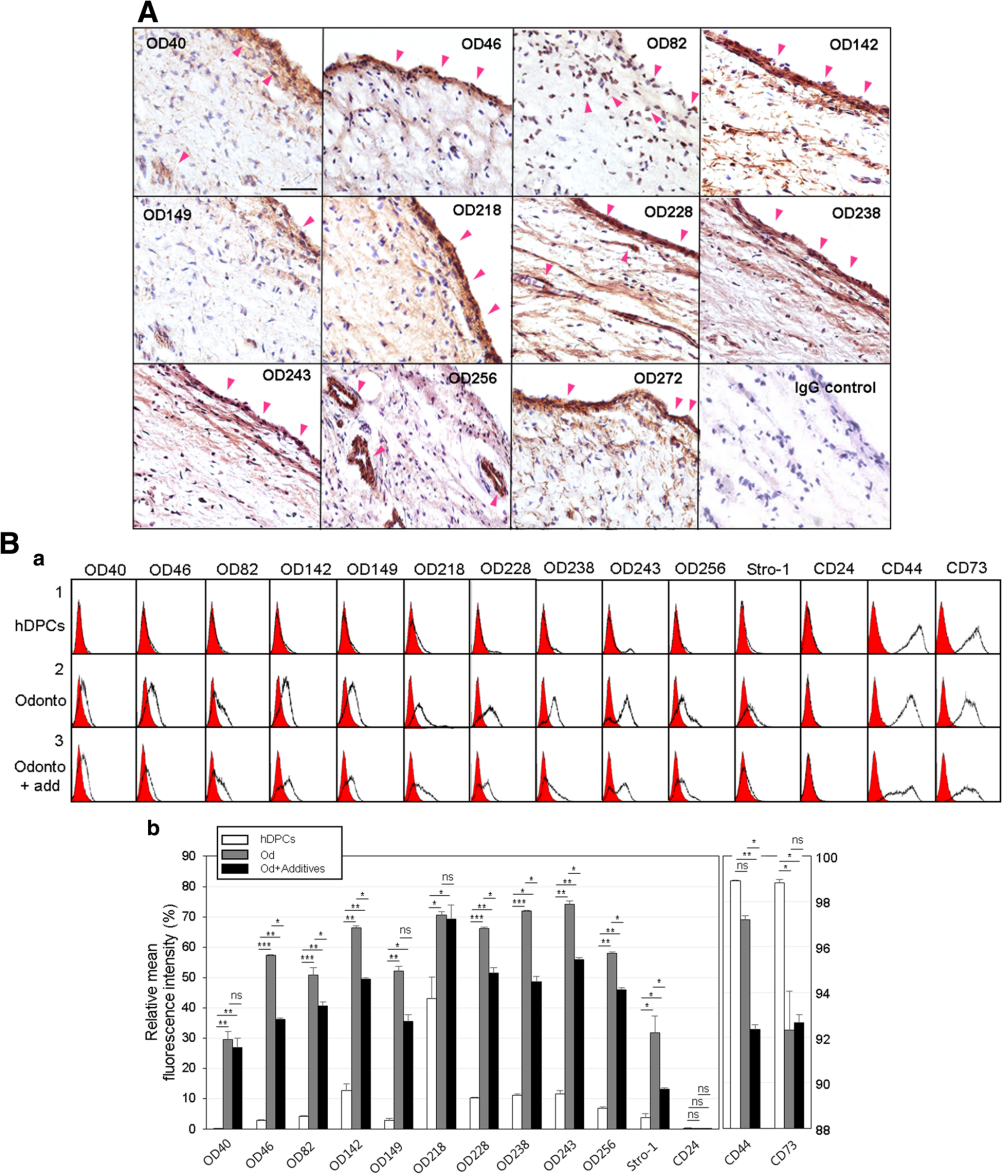
Cell surface binding of 12 antibodies is specific for the odontoblastic differentiation. **A** Immunohistochemistry of the antigenic molecules in tooth slices. The antigenic molecules recognized by 12 mAbs expressed in odontoblastic cell rich layer or perivascular region in pulp core (arrowheads). **B** Odontoblastic specificity of mAbs during the differentiation process. Three stages of odontoblastic differentiation were prepared as pre-odontoblast (hDPCs), odontoblast (hDPCs treated with BMPs, Od), and mature odontoblast (dentin forming cells, Od+Additives). Cell surface binding of mAbs on these cells were analyzed by immunocytometric analysis using by FACS (**a**). 1, hDPCs; 2, Od; 3, Od+Additives. Quantification of antibody binding was represented as mean fluorescence (**b**). Statistics were analyzed by Student’s *t* test, and asterisk indicated the significant difference between two samples. ***, *P* < 0.001; **, *P* < 0.01; *, *P* < 0.05; ns, not significant

The mAbs OD40, OD46, OD82, OD142, OD149, OD218, OD228, OD238, OD243, OD272, and OD278 mainly recognized the odontoblastic layer regions (Additional file 1: Figure S7, Od) of the dental pulp tissue in human tooth wherein odontoblasts were concentrated, and mAb OD256 strongly detected the perivascular regions in pulp core (Fig. 4A and Additional file 1: Figure S7, P). These results suggested that the mAbs identified were specific for odontoblastic cells in vivo. To confirm the specificity of these antibodies in the stage of the differentiation process, the binding affinity of mAbs were analyzed in hDPCs, in odontoblast-like cells, and in mature dentin-forming cells. Dentin maturation stage was induced by treatment with mineralization additives for 7 days. Although the expressions of Runx2, osteopontin, BSP, osteonectin, osteocalcin, and ALP increased gradually during maturation, those of collagen type-1, osterix, DMP-1, and DSPP, which were expressed in odontoblast-like cells specifically, decreased during mineralization (Additional file 1: Figure S8, gray and black bars in a). Stro-1, CD44, and CD73 are general surface markers for mesenchymal stem cells, and they were also expressed in dental stem cells [21]. Although Stro-1 expression was higher in odontoblast-like cells than in hDPCs, the expression of CD44 and CD73 was decreased in these cells. With dentinogenic maturation, the expression of Stro-1 and CD44 markers was much decreased (Fig. 4B, Stro-1, CD44, and CD73). The expression of CD24, a negative marker, was not detected in the cells at any stage (Fig. 4B, CD24). Cell surface binding of 12 mAbs was rarely detected in the undifferentiated hDPCs (Fig. 4B, panel 1 in a) and was dramatically increased in odontoblast-like cells derived from hDPCs after treatment with BMPs (Fig. 4B, panel 2 in a). After a 7-day induction of mineral formation, cell binding affinities of mAbs were decreased (Fig. 4B, panel 3 in a). Although surface expression of the OD40 and OD218 antigens was highly increased in odontoblast-like cells, its expression was remained stable during mineralization (Fig. 4B, OD40 and OD218 in a and b). These results indicated that mAbs identified in this experiment were useful to detect odontoblastic cytodifferentiation of hDPCs, and their binding affinities on the cell surface were decreased during mineral formation in dentinogenic maturation.

### Identification of antigenic molecules: candidates for odontoblast-specific cell surface and extracellular matrix molecules

**Fig. 5:**
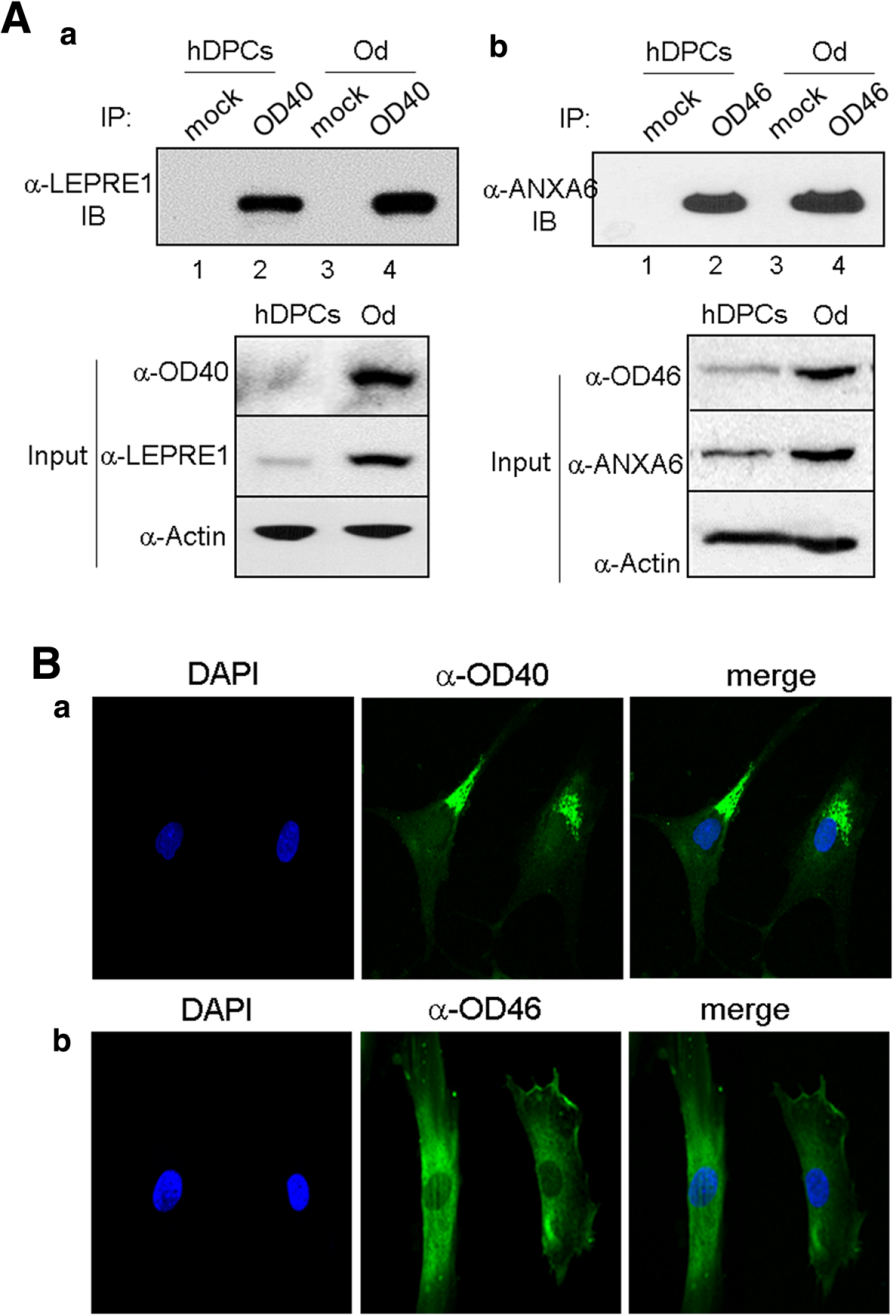
Antigen identification of two representative surface molecules recognized by mAb OD40 and OD46. **A** Antigenic molecules in the immnoprecipitants of OD40 (**a**) and OD46 (**b**) were applied to western blot (IB) with anti-leprecan-1 and anti-annexin A6 antibodies, respectively. Endogenous protein amounts were indicated in input panels. 1 and 3, IP with mouse IgG; 2 and 4, IP with mAbs. **B** Subcellular localization of the antigenic molecules against mAbs in the odontoblast-like cells. 1, nuclei stained by DAPI; 2, treatment with the primary mAbs and FITC-labeled secondary antibody; 3, merge with 1 and 2. Bar indicates as 100 μm

**Fig. 6:**
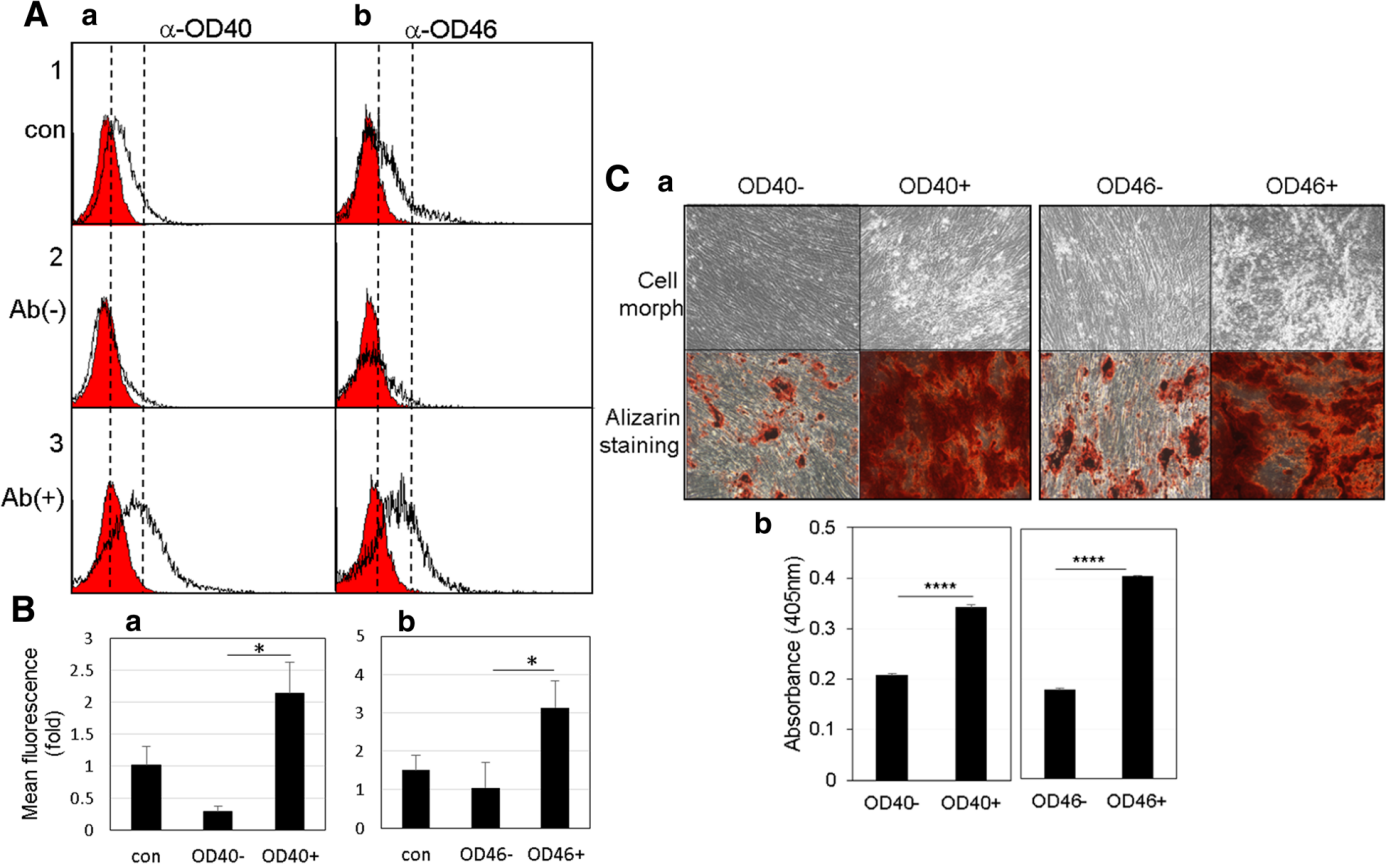
Cell sorting potentials of mAb OD40 and OD46. **A** The dental pulp cells are sorted by the magnetic-activated cell sorting system with anti-OD40 (**a**) and anti-OD46 (**b**) antibodies. After sorting, the antibody binding affinity was confirmed by FACS. **B** Relative mean fluorescence intensity was quantified by WinMDI program. 1, unsorted dental pulp cells; 2, antibody-negative cells; 3, antibody-positive cells. Cell population with significant antibody binding intensity was indicated as empty histograms. Statistics were analyzed by Student’s *t* test. *, *P* < 0.001. **C** Mineralization of OD40- and OD46-positive cells. Mineral deposits were stained by alizarin red (**a**), and were quantified (**b**) (see Additional file 1). Statistics were analyzed by Student’s *t* test, and asterisk indicated the significant difference between two samples. ****, *P* < 0.05

We first identified the antigenic molecules against the mAbs OD40 and OD46. The immunoprecipitates of OD40 and OD46 antigens were detected on SDS-PAGE by Coomassie blue staining. The bands of 180, 90, 55, and 50 kDa in OD40 immunoprecipitant and 70, 55, 50, and 25 kDa in OD46 immunoprecipitant were detected, and their amino acid sequences were analyzed by tandem mass spectrometry (Additional file 1: Figure S9 & S10, arrowheads and asterisks in a). As a result of tandem mass analysis, the 90 kDa and 70 kDa molecules were found to be leprecan-1 and annexin-A6/Calelectrin as the specific antigens of OD40 and OD46 antibodies, respectively (Additional file 1: Figure S9 & S10, b). The other bands were found to be the mouse IgG. The OD40 antigen leprecan-1 is a basement membrane-associated chondroitin sulfate proteoglycan, which is involved in the collagen secretory pathway of cells [26]. The OD46 antigen annexin A6/Calelectrin is a member of the annexin family, which is involved in many aspects of cellular membrane dynamics and in the regulation of membrane-associated proteins [27]. Indeed, anti-leprecan-1 and anti-annexin A6 antibodies showed strong cross-activity to the antigenic molecules OD40 and OD46 (Fig. 5A, a and b). As expected, the endogenous amounts of these molecules increased more in odontoblast-like cells than in hDPCs (Fig. 5A, input in a and b). By immunofluorescence analysis, the antigenic molecules against mAbs identified were localized to the cell surface or inner membrane structures (Fig. 5B). In addition to OD40 and OD46, other representative antigenic molecules also were detected in the similar subcellular localization patterns (Additional file 1: Figure S11). These data suggested that these antigenic molecules are the candidates of cell membrane and extracellular matrix components of odontoblast-like cells. To investigate the sorting potential of two antibodies, unsynchronous hDPCs and odontoblasts were separated by magnetic-activated cell sorting technique using these mAbs, and the antibody-cell binding affinities were confirmed through FACS analysis. A significant antibody binding disappeared in the fractions of flow-through (Fig. 6A, panel 2 in a and b). Based on quantification, the relative mean fluorescence intensities of the mAb OD40- and OD46-positive cells were increased in 7.5 and 3.1 times than in the negative cells, respectively (Fig. 6, panel 3 in A and B). Mineralization efficiency was highly increased in 1.75 and 2.35 times in the OD40- and OD46-positive cells, respectively (Fig. 6C).

## Discussion

In adult tissues, stem cells are usually in a dormant state and divide and generate a progeny of undifferentiated cells to retain their stemness by self-renewal. Stem cells live in a specialized microenvironment, called “niche,” which is highly specialized for each type of stem cell [28]. Niches comprise stem cells, supportive stromal cells, and the extracellular cellular matrix (ECM) wherein the stem cells are located [29]. They support stem cell survival and regulate stem cell behaviors, such as quiescence, self-renewal, and differentiation. The niche components influence the stem cell behavior and the interactions between stem cells and their niche are reciprocative, since stem cells are able to remodel the niche and secrete ECM components. Interactions between ECM and stem cells are directly mediated by numerous cell receptors, including integrins and other cell membrane molecules. These are the key heterodimeric transmembrane receptors involved in the connection of the extracellular environment to the intracellular cytoskeleton, thus mediating cell migration, proliferation, survival, and differentiation. Since integrins and other cell membrane molecules can also regulate signaling pathways in response to growth factors and cytokines [30] and signaling pathways can conversely regulate integrin expression, the specific activities of these ECM receptors are required to determine a particular type of stem cells [31]. Dental pulp stem cells derived from adult dental pulp tissue are reportedly a suitable cell source for regenerative dentistry. In dentin-pulp regeneration, it is important to enhance the odontoblastic differentiation potential of DPSCs. BMPs (e.g., BMP2, BMP4, and BMP7), TGFβ-1, FGFs, and VEGF have long been known to be incorporated into dentinogenesis in several previous reports [32, 33], and in this study, the combination treatment of BMP2 and BMP4 was the most effective in the induction of odontoblast-like cells from hDPCs (Fig. 1). Although cytokines for odontogenic induction are relatively well known, little was known about cell surface molecules and ECM for odontogenic niches. Two ECM molecules, DMP-1 and MEPE, are known as key molecules that play a regulatory role in the odontogenic induction of DPSCs [34, 35]. In this study, we focused on the identification of novel odontoblast-specific cell surface molecules, which can be present in the odontogenic environment. Thus far, for identifying cell surface markers, proteomic approaches coupled with mass spectrometry have generally been used [36, 37]. In this study, we performed decoy immunization using intact odontoblast-like cells because this method has the advantage of immediate providing specific antibodies useful for direct recognition of the cell surface molecules. We showed that 12 mAbs of IgG type obtained from 342 hybridomas specifically bound to odontoblast-like cells but not to hDPCs or osteoblasts (Figs. 2 and 4). Indeed, two of them tuned out to be a cell surface protein and an ECM component, and they specifically recognize in odontoblastic differentiation state (Fig. 5). In addition to OD40 and OD46, other antigenic molecules were localized in cell surface and inner secretory structures (Additional file 1: Figure S9). Taken together, we need further functional analysis, and the knowledge of these antigens can be useful in future studies on the regulation of cytodifferentiation of hDPCs and for understanding specific niches inducing odontogenic differentiation. More directly, mAbs can be applied to the detection and separation of odontoblastic cells from pulp tissue or undifferentiated hDPCs. Finally, these results suggest the immense potential of the construction of the effective odontogenic scaffolds using these cell surface/ECM-related antigens.

## Conclusions

This study has conducted a screening of human odontoblast-specific cell surface monoclonal antibodies (mAbs) through decoy immunization with intact odontoblast-like cells. For this, human odontoblasts were obtained from adult dental pulp cells through dentinogenic differentiation by co-treatment with BMP2 and BMP4. These mAbs accumulated in the odontoblastic layer and perivascular region in the pulp core in the tooth. Antigenic expression increased during odontogenic cytodifferentiation and had the potential to be components for membrane and extracellular matrix. These mAbs will be helpful for detecting odontoblasts and for providing information on cell adhesion and microenvironment during dentinogenesis.

## Additional file

Additional file 1: Additional Information for Materials and Methods. Table S1. Oligonucleotide primers used for antibody sequencing. Table S2. Oligonucleotide primers used for quantitative real-time PCR. Figure S1. Expression patterns of odontogenic and osteogenic markers by co-treatment with BMP2 and BMP4. Figure S2. BMP2 and/or BMP4 stimulation activate the Smad1/5/9 signal pathway. Figure S3. Osteogenic/dentinogenic maturation efficiency is enhanced in hDPCs treated with BMP2 and BMP4. Figure S4. Osteogenic/dentinogenic maturation efficiency is enhanced in hDPCs treated with BMP2 and BMP4. Figure S5. Odonto/osteoblastic marker expressions in hPDLCs. Figure S6. Cell surface bindings of 12 monoclonal antibodies on MG63 (a) and Saos-2 (b). Figure S7. Structure of human tooth. Figure S8. Odonto/osteoblastic marker expressions during the differentiation process. Figure S9. Antigen identification of surface molecules recognized by mAb OD40. Figure S10. Antigen identification of surface molecules recognized by mAb OD46. Figure S11. Subcellular localization of the antigenic molecules against the representative mAbs in the odontoblast-like cells. (DOCX 3604 kb)

## Abbreviations

ALP: Alkaline phosphatase
BMP: Bone morphogenic proteins
BSP: Bone sialoprotein
DMP-1: Dentin matrix protein-1
DSPP: Sialophosphoprotein
ECM: Extracellular matrix
hDPCs: Human dental pulp cells
hFOB: Human fetal osteoblast
hPDLCs: Human periodontal ligament cells
mAb: Monoclonal antibody
SDS-PAGE: Sodium dodecyl sulfate-polyacrylamide gel electrophoresis
