# Supplementary material for: Identification of dentinogenic cell-specific surface antigens in odontoblast-like cells derived from adult dental pulp
Source: Stem Cell Res Ther. 2019 Apr 27;10:128. doi: 10.1186/s13287-019-1232-y (PMC6487011; doi:10.1186/s13287-019-1232-y)
Supplement: Supplementary file 1 — Additional Information for Materials and Methods. Table S1. Oligonucleotide primers used for antibody sequencing. Table S2. Oligonucleotide primers used for quantitative real-time PCR. Figure S1. Expression patterns of odontogenic and osteogenic markers by co-treatment with BMP2 and BMP4. Figure S2. BMP2 and/or BMP4 stimulation activate the Smad1/5/9 signal pathway. Figure S3. Osteogenic/dentinogenic maturation efficiency is enhanced in hDPCs treated with BMP2 and BMP4. Figure S4. Osteogenic/dentinogenic maturation efficiency is enhanced in hDPCs treated with BMP2 and BMP4. Figure S5. Odonto/osteoblastic marker expressions in hPDLCs. Figure S6. Cell surface bindings of 12 monoclonal antibodies on MG63 (a) and Saos-2 (b). Figure S7. Structure of human tooth. Figure S8. Odonto/osteoblastic marker expressions during the differentiation process. Figure S9. Antigen identification of surface molecules recognized by mAb OD40. Figure S10. Antigen identification of surface molecules recognized by mAb OD46. Figure S11. Subcellular localization of the antigenic molecules against the representative mAbs in the odontoblast-like cells. (DOCX 3604 kb) [file 13287_2019_1232_MOESM1_ESM.docx]

**Additional Information for Materials and Methods**

***Antibody purification***

The antibody was purified by column chromatography from hybridoma culture media. Cell media were loaded on the Protein G-agarose column (Incospharm) for IgG mAb purification. After washing with PBS, the bound IgG were eluted with 100 mM glycine buffer (pH2.8) and then neutralized by 1 M Tris-HCl buffer (pH9.0). After dialysis in PBS at 4℃, antibody was quantified.

***Alizarin-red S staining and alkaline phosphatase (ALP) activity***

Cells were fixed with 4% paraformaldehyde for 15 min, washed with PBS, and treated with 2% alizarin red S (pH4.5, Sigma). For quantification, plate was incubated with 10% acetic acid for 30 min at RT and heated for 10 min at 85℃. Then, supernatants were neutralized by 10% ammonium hydroxide. ALP activity was analyzed using Alkaline Phosphatase Activity Colorimetric assay kit (BioVision) according to the supplier’s protocol. 1x10^5^ cells were detached from culture dishes by using enzyme-free dissociation buffer and lysed in the Assay buffer by sonication. Cell lysates were incubated with 5 mM *p*-nitrophenyl phosphate for 60 min at 25℃ with blocking out the light. For quantification, absorbance values of analytic samples from alizarin staining and ALP assay were measured at 405 nm using a Multi-Detection Microplate reader (Molecular Devices).

**Additional Information for Tables and Figures**

Additional file 1

**Table S1.** Oligonucleotide primers used for antibody sequencing

| **Target** | **Primer** | **Sequences** |
| --- | --- | --- |
| Variable region of Heavy chain | For-VH1 | 5’- gaattcSARGTNMAGCTGSAGSAGTC |
|  | For-VH2 | 5’-gaattcSARGTNMAGCTGSAGSAGTCWGG |
|  | Rev-IgG1 | 5’-gtcgacATAGACAGATGGGGGTGTCGTTTTGGC |
|  | Rev-IgG2b | 5’-gtcgacAGGGGCCAGTGGATAGACTGATGG |
| Variable region of Light chain | For-LVκ | 5’-aagcttGAYATTGTGMTSACMCARWCTMCA |
|  | Rev-LCκ | 5’-gtcgacGGATACAGTTGGTGCAGCATC |

*Base degeneracies were indicated as: S, C/G; R, A/G; N, A/T/G/T; M, A/C; W, A/T; Y, C/T

**Table S2.** Oligonucleotide primers used for quantitative real-time PCR

| **Target gene** | **Forward primer** | **Reverse primer** |
| --- | --- | --- |
| Scleraxis | 5’-AGAAAGTTGAGCAAGGACC | 5’-CTGTCTGTACGTCCGTCT |
| Runx2 | 5’-GTCTCACTGCCTCTCACT | 5’-TACACACATCTCCTCCCTTC |
| hCOLα1 | 5’-GGAGGAGAGTCAGGAAGG | 5’-TCAGCAACACAGTTACACAA |
| Osteopontin (ONT) | 5’-CTGTTGCCTGTCTCTAAACC | 5’-CACCATCATCAAATTCTCCT |
| Ostrerix | 55’-TTGACATGTACCCCTTTCTG | 5’-AATACCCCTGATGAAGAGG |
| DMP-1 | 5’-ACTCTCAAGAAGACAGCAA | 5’-GACTCACTCACCACCTCT |
| Bone sialoprotein (BSP) | 55’-ACCGAGCCTATGAAGATGA | 5’-CTTCCTGAGTTGAACTTCGA |
| DSPP | 5’-CAGTACAGGATGAGTTAAATGCCAGTG | 5’-CCATTCCCTTCTCCCTTGTGAC |
| GAPDH | 5’-GTATGACAACAGCCTCAAGAT | 5’-CCTTCCACGATACCAAAGTT |
| Osteonectin (ONT) | 5’-CTGTTGCCTGTCTCTAAACC | 5’-CACCATCATCAAATTCTCCT |
| Osteocalcin (OCN) | 5’-TGAGTCCTGAGCAGCAG | 5’-TCTCTTCACTACCTCGCT |
| Alkaline Phosphatase (ALP) | 5’-CTTGACCTCCTCGGAAGACACTC | 5’-CGCCCACCACCTTGTAGCC |

**
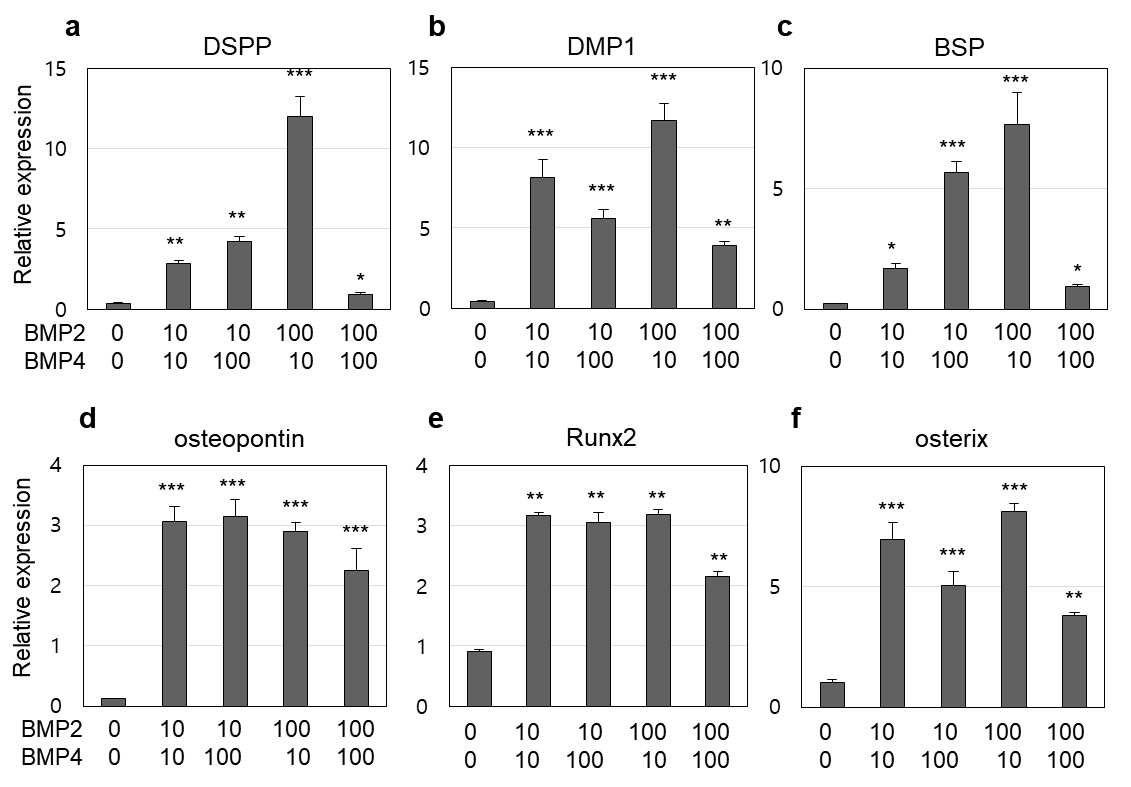
**

**Figure S1**. Expression patterns of odontogenic and osteogenic markers by co-treatment with BMP2 and BMP4. hDPCs were treated with the indicated concentrations of BMP2 and BMP4. Bar graphs represented the mean of three independent experiments±SD. Statistical data were analyzed by Student’s *t*-test, and asterisk indicated the significant difference. ***, P<0.001; **, P <0.05; *, P <0.01; ns, not significant.


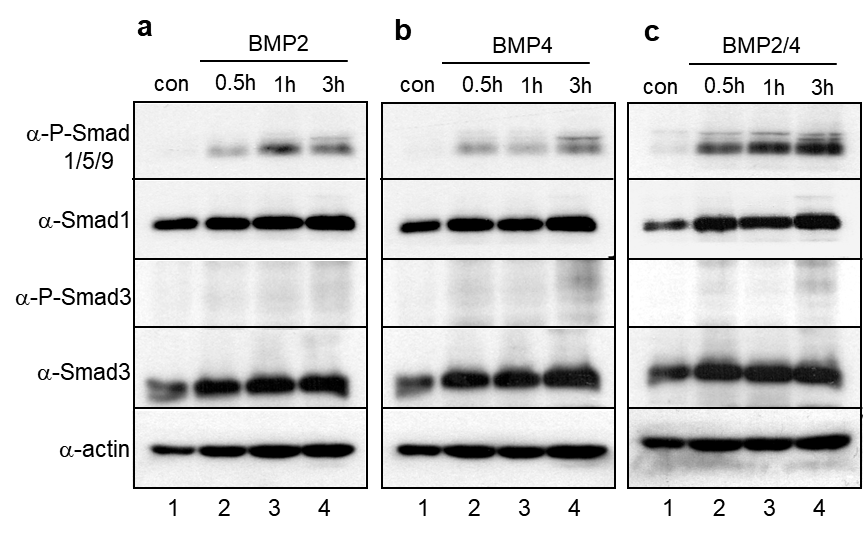


**Figure S2**. BMP2 and/or BMP4 stimulation activate the Smad1/5/9 signal pathway. a, treatment with 100 ng/ml BMP2; b, treatment with 10 ng/ml BMP4; c, co-treatment with 100 ng/ml BMP2 and 10 ng/ml BMP4. After indicated times of treatment, hDPCs were harvested and total cell extracts were introduced in western blot analysis. Actin used as the normalization control.


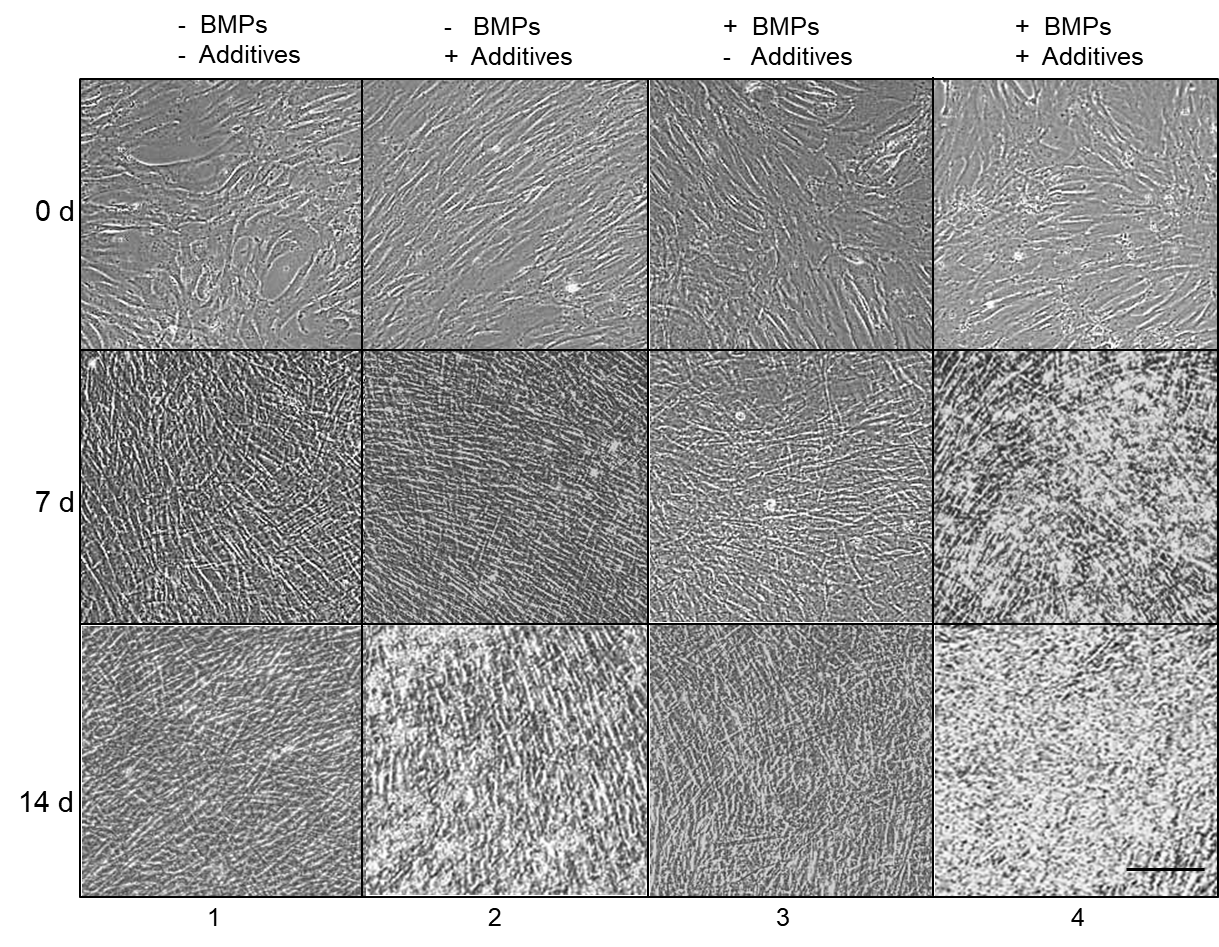


**Figure S3**. Osteogenic/dentinogenic maturation efficiency is enhanced in hDPCs treated with BMP2 and BMP4. To induce mineral formation, cells were cultured in media containing mineralization additives for the indicated time, following BMPs treatment. Mineral formation on the cell surfaces was investigated by direct observation under the microscope. -BMPs/-Additives, hDPCs untreated; -BMPs/+Additives, hDPCs incubated in media containing mineralization additives; +BMPs/-Additives, hDPCs treated with BMPs for odontogenic differentiation; +BMPs/+Additives, hDPCs incubated in media containing mineralization additives following BMPs treatment.


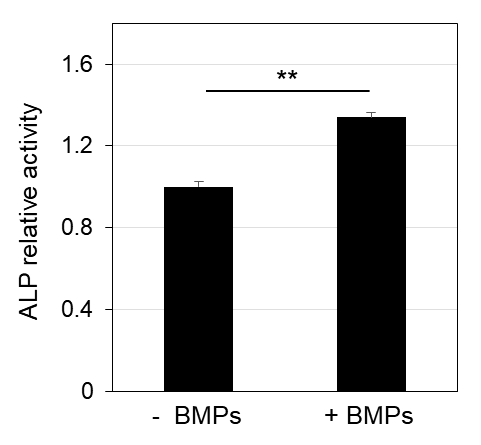


**Figure S4**. Osteogenic/dentinogenic maturation efficiency is enhanced in hDPCs treated with BMP2 and BMP4. To induce mineral formation, cells were cultured in media containing mineralization additives for the indicated time, following BMPs treatment. Alkaline phosphatase activities in hDPCs (-BMPs) and odontoblast-like cells (+BMPs), which were incubated in Additives for 7 days. Statistical data were analyzed by Student’s t-test, and asterisk indicated the significant difference between two samples. **P <0.05.


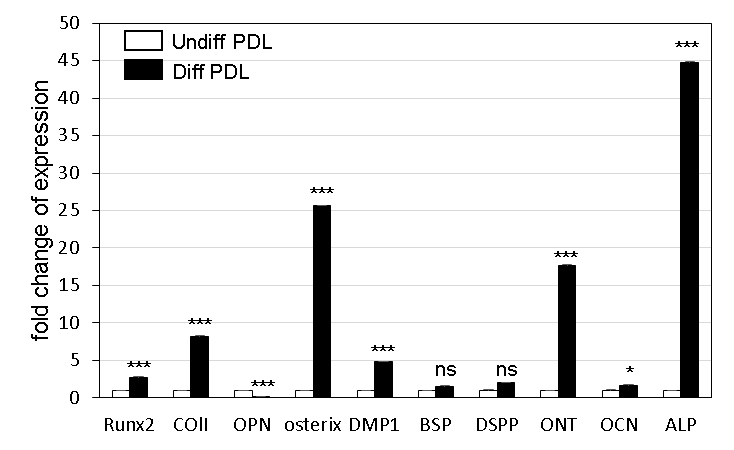


**Figure S5**. Odonto/osteoblastic marker expressions in hPDLCs. hPDLCs were treated with 100ng/ml BMP2 for 7days, and confirmed the cell stages by odonto/osteoblastic marker expressions. White bars, untreated hPDLCs; black bars, BMP2 treated hPDLCs. Statistics were analyzed by Student’s t-test, and asterisk indicated the significant difference between two samples. ***, P<0.001; **, P <0.01; *, P <0.05; ns, not significant.


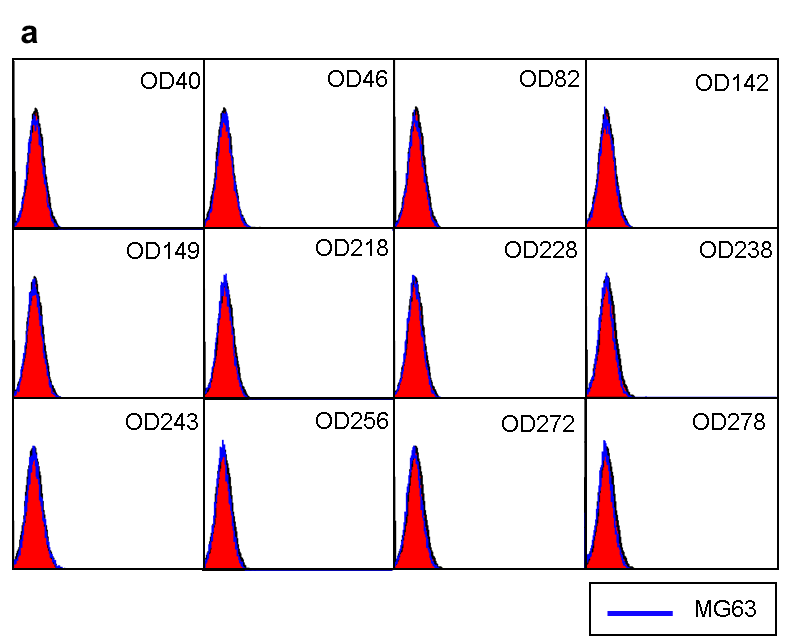


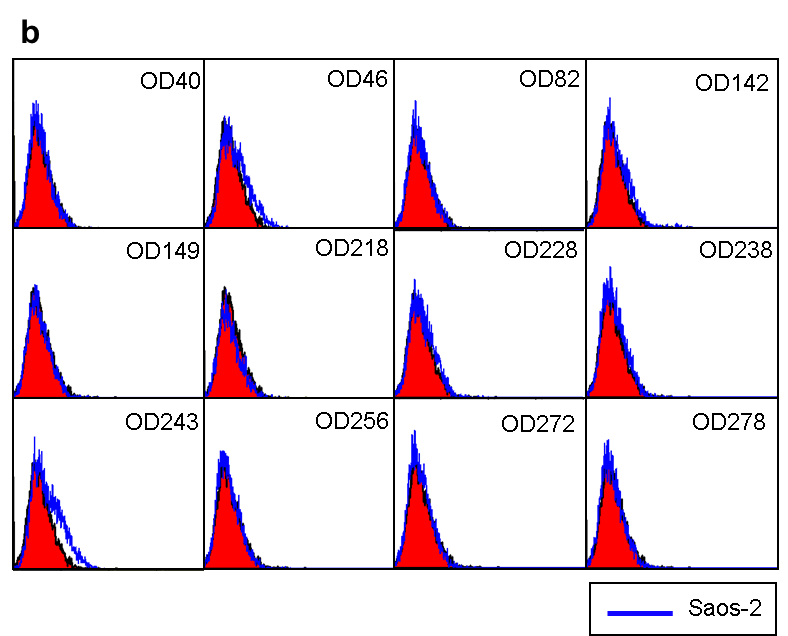


**Figure S6**. Cell surface bindings of 12 monoclonal antibodies on MG63 (a) and Saos-2 (b). The interaction between each antibody and cells were analyzed by immunocytometric analysis using by FACS


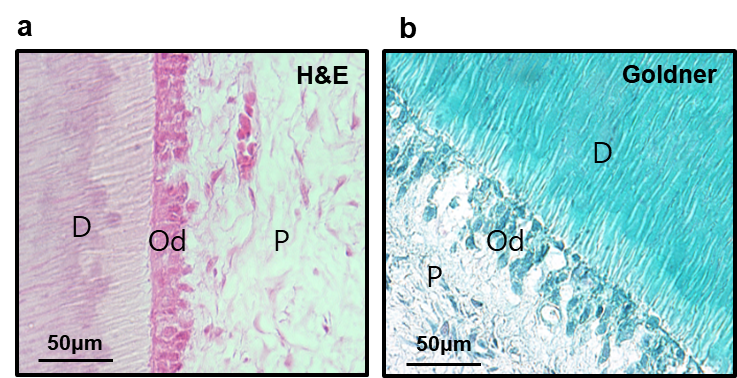


**Figure S7**. Structure of human tooth. Parts of dentin (indicated by D), odontoblast cell layer (indicated by Od), and pulp core (indicated by P) were discriminated by hemtoxylin/eosin (a) or Goldner (b) staining.


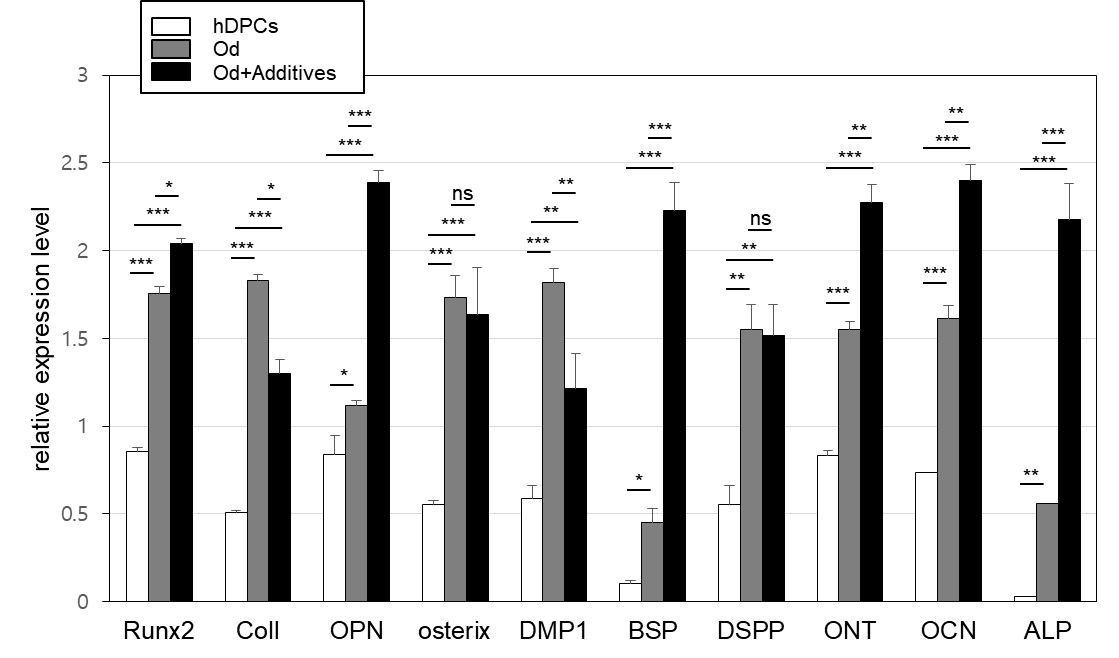


**Figure S8**. Odonto/osteoblastic marker expressions during the differentiation process. Three stages of odontoblastic differentiation were prepared as pre-odontoblast (hDPCs), odontoblast (hDPCs treated with BMPs, Od), and mature odontoblast (dentin forming cells, Od+Additives), and confirmed the cell stages by odonto/osteoblastic marker expressions. White bars, hDPCs; grey bars, Od; black bars, Od+Additives. Statistics were analyzed by Student’s t-test, and asterisk indicated the significant difference between two samples. ***, P<0.001; **, P <0.01; *, P <0.05; ns, not significant.


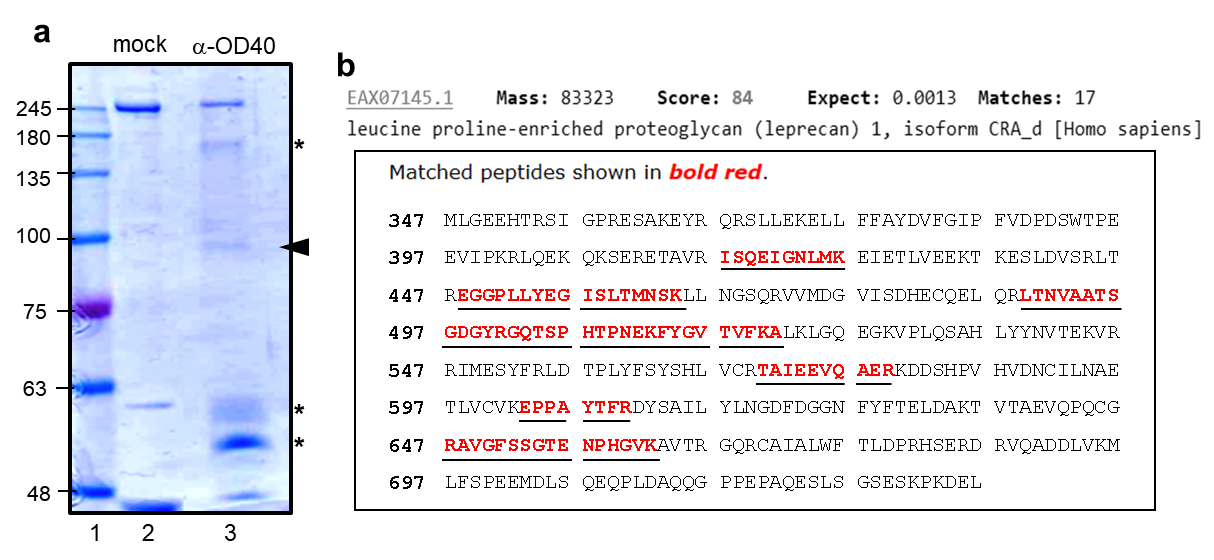


**Figure S9**. Antigen identification of surface molecules recognized by mAb OD40. Total lysates originated from 5x10^7^ of odontoblast-like cells were subjected into immunoprecipitation, and were analyzed on SDS-PAGE. Antigenic molecules in the immnoprecipitants were visualized by Coomassie-Blue staining (a), and detectable bands were identified by tandem mass spectrometry (b). Antigenic molecules and antibodies used for IP were indicated by arrowheads and asterisks, respectively.


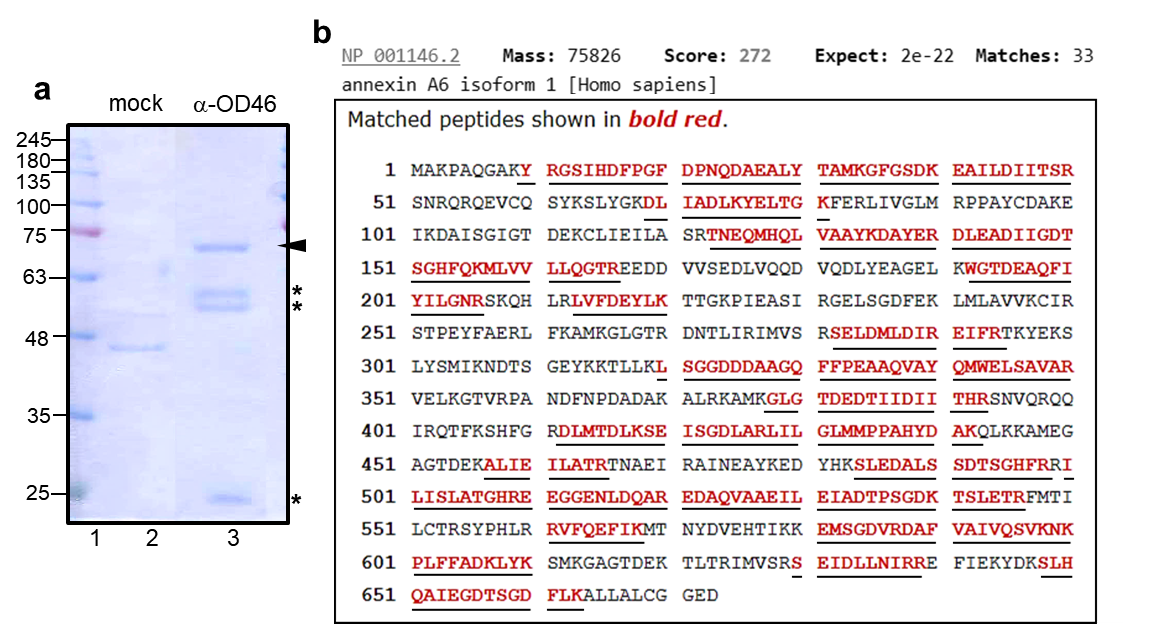


**Figure S10**. Antigen identification of surface molecules recognized by mAb OD46. Total lysates originated from 5x10^7^ of odontoblast-like cells were subjected into immunoprecipitation, and were analyzed on SDS-PAGE. Antigenic molecules in the immnoprecipitants were visualized by Coomassie-Blue staining (a), and detectable bands were identified by tandem mass spectrometry (b). Antigenic molecules and antibodies used for IP were indicated by arrowheads and asterisks, respectively.


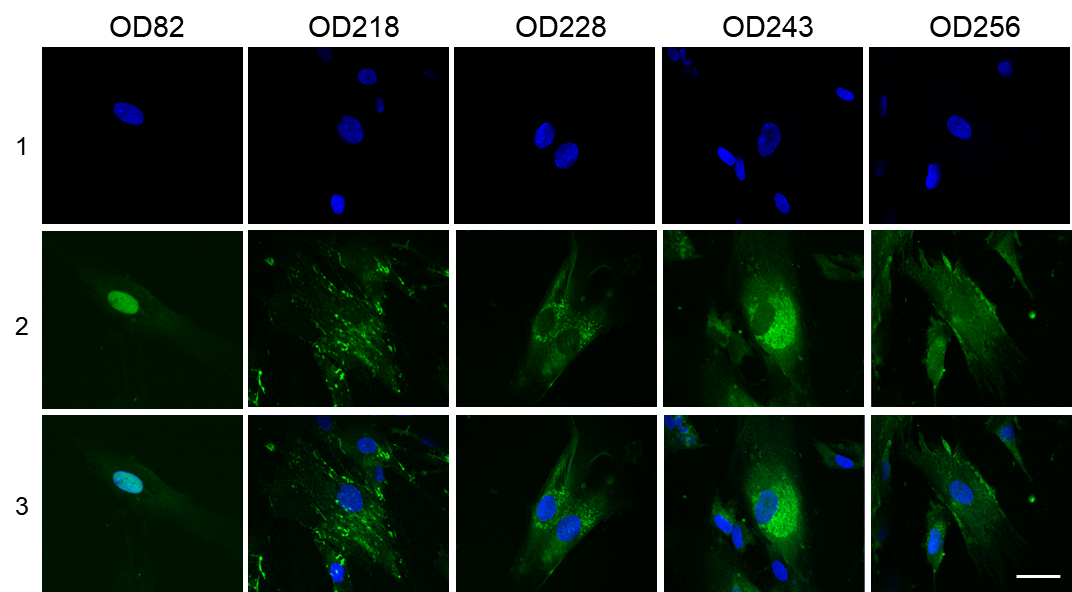


**Figure S11**. Subcellular localization of the antigenic molecules against the representative mAbs in the odontoblast-like cells. 1, nuclei stained by DAPI; 2, Treatment with the primary mAbs and FITC-labeled secondary antibody; 3, merge with 1 & 2. Bar indicates as 100-μm.
